# Advances in Bacteriophage-Mediated Control of Plant Pathogens

**DOI:** 10.1155/2012/326452

**Published:** 2012-08-13

**Authors:** Rebekah A. Frampton, Andrew R. Pitman, Peter C. Fineran

**Affiliations:** ^1^Department of Microbiology & Immunology, University of Otago, P.O. Box 56, Dunedin 9054, New Zealand; ^2^New Zealand Institute for Plant & Food Research, Private Bag 4704, Christchurch 8140, New Zealand

## Abstract

There is continuing pressure to maximise food production given a growing global human population. Bacterial pathogens that infect important agricultural plants (phytopathogens) can reduce plant growth and the subsequent crop yield. Currently, phytopathogens are controlled through management programmes, which can include the application of antibiotics and copper sprays. However, the emergence of resistant bacteria and the desire to reduce usage of toxic products that accumulate in the environment mean there is a need to develop alternative control agents. An attractive option is the use of specific bacteriophages (phages), viruses that specifically kill bacteria, providing a more targeted approach. Typically, phages that target the phytopathogen are isolated and characterised to determine that they have features required for biocontrol. In addition, suitable formulation and delivery to affected plants are necessary to ensure the phages survive in the environment and do not have a deleterious effect on the plant or target beneficial bacteria. Phages have been isolated for different phytopathogens and have been used successfully in a number of trials and commercially. In this paper, we address recent progress in phage-mediated control of plant pathogens and overcoming the challenges, including those posed by CRISPR/Cas and abortive infection resistance systems.

## 1. Introduction

In October 2011 the United Nations announced that the global human population had reached 7 billion. The world is facing not only this increase in population, but also a decrease in land availability for agriculture and a changing climate [1]. It is apparent that there is a requirement to increase food production to feed the growing population and a need to achieve this with diminished land and water resources [1]. A major threat to food production is plant diseases, which are influenced by changing agricultural practices and more global trade [2]. Recent topical examples include citrus greening of oranges caused by psyllids that transmit bacteria belonging to the genus *Candidatus *Liberibacter [3] and canker of kiwifruit caused by the bacterium *Pseudomonas syringae* pv. *actinidiae *[4]. Citrus greening has doubled the cost of orange production for growers in Florida, where the disease was first identified in 2005 [3]. In New Zealand, where *Pseudomonas syringae* pv. *actinidiae* was discovered in late 2010, 40% of orchards have been infected resulting in a significant economic cost to the industry [5]. 

A variety of approaches are required to minimise the impact of bacterial plant diseases on the quantity, quality, and economy of food production. Conventional control measures involve the implementation of operating practices to prevent further infections, removal of infected plant tissue, and appropriate disposal to stop the transmission of the pathogen from one site to another. Other methods to control phytopathogens include chemicals such as pesticides, to control insect vectors, antibiotics (e.g., tetracycline and streptomycin), and copper. Copper has been used for over 100 years and antibiotics such as streptomycin have been used since the 1950s [6, 7]. Streptomycin has been used for many years for the control of pathogens, including *Pseudomonas syringae *pathovars, and resistance has been regularly reported following use [6–8]. Another concern with antibiotics is the spread of resistance genes to other bacteria, including other pathogens or nonpathogenic bacteria present in the environment [7]. Copper resistance has also been documented for plant pathogens, including *Xanthomonas *and *Pseudomonas *species [9–11]. Continual use of copper sprays can lead to toxic levels in the environment [12, 13]. Therefore, it is favourable to replace or integrate chemical control methods with less toxic biological methods. 

There is mounting interest in using bacteriophages (phages) as biocontrol agents (BCAs) to target phytopathogens. Phages are viruses that specifically infect bacteria, yet have no direct negative effects on animals or plants. Infection of a bacterium by a virulent phage typically results in rapid viral replication, followed by the lysis of the bacterium and the release of numerous progeny phages. These phages can then proceed to infect neighbouring bacteria. Therefore, the numbers of phage will expand when target pathogens are encountered and the therapy will essentially be amplified in response to the bacterial infection. This is a distinct advantage over other treatments, such as antibiotics. Since the 1920s, within a decade of the first discovery of phages, their potential as therapeutic agents for use in agriculture was under investigation and provided some promising results (see [14] for review of the early literature). Recent years have seen a resurgence of interest in phage therapy for the control of phytopathogens (see [15–17] for recent reviews). In part, this renewed interest is due to the nontoxic nature of phages and their ability to infect antibiotic or heavy metal resistant bacteria. Successful phage therapy is being applied commercially to processed and packaged foods by Intralytix and Micreos Food Safety (formerly EBI Food Safety) and to agricultural crops by Omnilytics. There is also interest in the use of phage in the detection of phytopathogens. Indeed, many of the first phages against plant pathogens were isolated for diagnosis and strain typing [18] and recently genetic advances are yielding effective phage-based reporter systems [19, 20]. In this paper we will examine the use of phage as BCAs for bacterial plant diseases. First, we will address the initial isolation and laboratory characterisation of phages. Next, we will discuss the transition from *in vitro* analyses to bioassays and field/greenhouse trials to commercialisation and application. Finally, we will provide an analysis of phage resistance in phytopathogens and address how this can be avoided or minimised when developing phage BCAs. 

## 2. Initial Phage Characterisation

### 2.1. Isolation and Host Range of Phages

The initial stage in developing a phage-based BCA involves the isolation of phages. Phages can be isolated relatively simply from soil, water, and plant material collected from multiple locations using the soft agar overlay technique and a range of host pathogens. Isolation methods are well established and covered in several reviews [21, 22], and many successful studies have resulted from this approach (e.g., [23–25]). To isolate diverse phages it is important to include a range of host bacterial strains that represent the diversity of the pathogens involved in the disease [17, 26]. Phages that produce clear plaques should be chosen preferentially to reduce the isolation of temperate phages as certain temperate phage can cause lysogenic conversion, a process whereby virulence genes carried by the prophage contribute to pathogenicity of the bacterial lysogen [27]. This approach increases the isolation of phages from the order Caudovirales (Myoviridae, Siphoviridae, and Podoviridae). Filamentous phage from the Inoviridaefamily produce a chronic infection that results in the continuous release of phage from growing bacterial cells. Filamentous phage could also be a BCA option and this is discussed in more detail in Section 3.5 [28].

Determining the host range of each phage enables the design of a cocktail capable of infecting all known pathogenic strains involved in the disease. Although the host range of most phages is usually narrow, bacteria isolated from the plant environment should be tested for lysis by the putative BCA phages to ensure a minimal impact of the phages on the wider microbial community and potential commensal strains.

### 2.2. Basic Characterisation of Phages

Once isolated, the phages need to be characterised to ensure they are appropriate BCAs. This information will allow for rational design of a phage cocktail and enable the tracking of phages during bioassays and field trials. Transmission electron microscopy and molecular methods, such as restriction pattern analysis of phage DNA, enable assessment of phage diversity. Identification of the phage receptors can assist in the rational selection of phages that target through different mechanisms to reduce the frequency of resistance (discussed in Section 5).

The development of next-generation sequencing has dramatically reduced the cost of determining the complete DNA sequence of a phage genome. The sequence also enables the design of quantitative PCR strategies for phage tracking in field trials [26] and if present, genes required for integration/lysogeny or that encode known toxins, antibiotic resistance, or virulence factors can be identified. Phage-mediated horizontal transfer of bacterial genes by specialised and generalised transduction should be avoided [27]. The possibility of specialised transduction is eliminated by removing temperate phages but assays are necessary to examine generalised transduction. These assays cannot rule out transduction but enable the identification and elimination of phages with a high transducing frequency [27]. It has been proposed that if a phage cocktail is used then any bacteria that receive additional DNA through transduction can still be killed by another phage in the mixture [17]. An understanding of the basic growth parameters of the phage will aid in the design of a phage BCA. Therefore, investigating the length of infection and burst size with one-step growth curves at temperatures and conditions likely to be encountered in the field iss important. It is also important to identify conditions under which the phage can be successfully stored [15]. Following phage characterisation the next step is to perform bioassays and/or field trials which are discussed in Section 3. 

### 2.3. Alternative Biocontrol Phage Technologies

Through phage genomics, genes encoding other potential biocontrol options have been identified. One example is phage endolysins, which have been investigated for use against antibiotic resistant bacteria that colonize human mucosal membranes [29]. Endolysins are phage-encoded peptidoglycan hydrolases that work in concert with holins to ensure cell lysis occurs following phage maturation [30]. Generally, the C-terminus binds the bacterial cell wall and positions the enzymatically active N-terminus close to its target. Due to their high level of specificity, phages CMP1 and CN77 were originally used for detection and identification of plant pathogenic *Clavibacter michiganensis* strains [31, 32]. *Clavibacter michiganensis* subsp. *michiganensis *is a recalcitrant tomato pathogen because there are no resistant cultivars and chemical control agents are ineffective. The endolysins of CMP1 and CN77 responsible for degrading the bacterial cell wall have been pursued as potential antimicrobials [33]. Sequence analysis revealed that the similarity of the catalytic domains of the CMP1 and CN77 endolysins was low, suggesting they target different covalent bonds within the peptidoglycan. However, both endolysins are specific for *Clavibacter michiganensis* subsp. *michiganensis*, as observed for the phages [34]. The use of endolysins is still highly specific but avoids concerns of using a replicating BCA. Compared with the application of whole phage preparations, the development of lysine-based therapies is more technically challenging. For example, the generation of lysins requires greater molecular insight than phage therapy because sequencing, identification, cloning, characterization, and purification of the lysins must be performed.

## 3. Trials of Phage Biocontrol

Once a selection of phytopathogen-specific phages is obtained and the initial characterisation has indicated their potential for biocontrol, the next step is to test their efficacy in relation to plant disease. First it is necessary to scale-up phage preparations, which requires knowledge about the characteristics and lifestyle of the phages [35]. To test phytopathogen control, a range of approaches have been taken, from laboratory-based bioassays through greenhouse and field trials.  Table 1 summarises some results of phage trials that have been performed on a range of phytopathogens including *A. tumefaciens*, *Dickeya solani*, *Pectobacterium carotovorum*, *Erwinia amylovora*, *Pseudomonas syringae*, *Ralstonia solanacearum*, *Streptomyces scabies*, and *Xanthomonas* species. Many of these studies have given promising results. However, a number of factors can contribute to phage biocontrol trials that have failed. Firstly, to understand the reason for success or failure it is important to measure the dynamics of phage and host, for example, via quantitative PCR or traditional enumeration assays [26]. Secondly, field trials are biologically complex and the presence of other microbes, including other pathogens, can influence the effectiveness of the phages. In some cases pathogens of a different genus or species will cause very similar disease symptoms but are not killed by the phages [23]. Therefore, it is important to check which organism(s) caused the disease symptoms and if it is the targeted bacterial host, whether phage-resistant mutants account for the lack of phage killing or not. It is important to confirm that phage preparations are free of any pathogenic bacteria used in the production process, which highlights the requirement for phage-only controls in trials. The absence of a “gold standard” positive treatment, where available, in some studies makes evaluating the efficacy less informative. Other factors including the type of water and the presence of certain components in some fertilizers can influence phage viability and the trial's success [36]. Despite these challenges, many of the studies in Table 1 demonstrated a positive effect of phage treatment.

Each phage-phytopathogen-plant system has unique features and requires characterisation and optimisation of the phage biocontrol. External and environmental factors can play a role and cause variable results. For example, greenhouse-grown crops are in a more stable environment, whereas plants grown outside are exposed to more variable weather conditions and these factors vary between geographic locations. Phage survival and their persistence at the required site of action are affected by conditions such as pH, temperature, desiccation, rain, and UV. The most damaging factor appears to be UV irradiation in sunlight [52]. Interestingly, some plant extracts negatively affect the growth or viability of phage *in vitro* [53, 54] but it is unclear whether this is relevant during phage therapy on plants. Various approaches to address these challenges in phage biocontrol are available and will be discussed in the following sections.

### 3.1. Protective Formulations and Application Timing

In order to minimise UV damage, some researchers have investigated protective formulations. Few published studies have addressed stabilising agents, but one group found that certain combinations of sucrose, Casecrete NH-400, pregelatinised corn flour or skim milk, increased the persistence of phages active against *Xanthomonas campestris *pv. *vesicatoria *in greenhouse and field trials and improved treatment efficacy [51]. However, in a trial with phages against *Xanthomonas axonopodis *pv.* citri *and *citrumelo*, skim milk inhibited the action of the phages even though the phages persisted on the leaf surface for longer [25]. Balogh et al. also investigated the effect of the time of day of phage application and demonstrated that evening applications increased phage survival [51]. The lower UV intensity at dusk was attributed to this improved viral longevity. The frequency of phage application also appears to be specific to the particular phage-phytopathogen-plant system and the best results vary from daily to weekly application [25, 44, 48, 50, 51, 55]. Therefore, these conditions must be optimised for each newly developed phage BCA.

### 3.2. Coapplication with Other Control Strategies

Researchers have begun examining the effects of combining phage with existing or new control measures. Hypersensitive response and systemic acquired resistance plant activators have been tested against *Xanthomonas *spp. in two disease models in the presence or absence of phages. The combined approach provided disease control equal to, or greater than, either treatment alone [44, 48, 49] (Table 1) and comparable results were obtained with phage and copper hydroxide with mancozeb [48]. Recently, it was demonstrated that lipid-containing phages were the most susceptible to copper, whereas most dsDNA phages were unaffected [56]. This indicates that dsDNA phages are the best candidates for plant disease therapies in combination with copper. Phages can also be used in combination with streptomycin as they are not a direct target of antibiotics [45]. Cotherapy with copper or streptomycin could enable a “belt and braces” approach to minimise streptomycin/copper or phage resistance. However, this approach is not possible in some parts of the world. For example, regulations that disallow the use of streptomycin for the control of plant diseases have been introduced in the EU [57]. Phage compatibility with other agrichemicals has not been thoroughly examined. Phages used would require testing for viability and persistence in the presence of any agrichemical treatment used simultaneously on the affected plants.

A range of bacterial and fungal BCAs that include pathogen antagonists have been developed and are commercially available. These include BlightBan A506 (*Pseudomonas fluorescens *A506; Nufarm Americas Inc), Blossom Bless (*Pantoea agglomerans *P10c; Gro-Chem NZ Ltd), and Superzyme (*Bacillus subtilis*, *Trichoderma*, and *Pseudomonas putida*; J H Biotech Inc). When used on their own, the protection provided by some of these products often varies and does not compare favourably with streptomycin, which is considered the benchmark [58]. These BCAs can complement conventional control strategies. For example, use of BCAs in the control of fire blight [59, 60] in apple trees reduced the number of streptomycin sprays required to provide the same amount of protection against *E. amylovora *infection in apple blossoms [58]. Growth of these BCAs is thought to be required for their action as competitors. Variable environmental conditions influence growth and the regulation of the production of secondary metabolites, such as antibiotics, which might account for some of the inconsistent responses to BCAs. One approach to improve plant protection is to incorporate phages into existing BCA products as part of the pest management plan. 

### 3.3. Use of Carrier Bacteria

The abundance of host bacteria, in addition to the environmental factors mentioned above, causes fluctuations in phage numbers [61]. To improve phage persistence and efficacy the idea of coapplication of phages with nonpathogenic host bacteria to support phage replication has been raised [26, 62]. It is necessary to identify a suitable nonpathogenic carrier bacterium that does not affect the plant and to isolate phages that infect both the target phytopathogen and the carrier strain. Therefore, isolation of broad host range phages can also be beneficial for the purposes of biological control. Care is needed to use phages that do not infect plant beneficial bacteria in the phyllosphere or rhizosphere. 

The use of competitive antagonists has been investigated in the control of fire blight for many years and can prevent or reduce infection [24, 26, 63]. An extensive panel of *Erwinia amylovora *phages have been isolated and characterised [24, 38, 39, 62, 64] and their use with carrier bacteria investigated [17, 26, 62]. In an early study a temperate phage that infected both a saprophyte and *Erwinia amylovora* provided increased protection in a pear slice bioassay [62]. However, current opinion is that temperate phages should be avoided for phage therapy. Recently, a *Pantoea agglomerans* carrier system was developed which amplified the phages and reduced disease in blossom bioassays [17, 26]. The application of lytic phages against *Erwinia amylovora* in conjunction with the *Pantoea agglomerans *carrier strain to potted apple trees reduced the incidence of fire blight to the same level as streptomycin [24]. Application of the carrier strain itself also helped prevent infection. In orchard trials, the coapplication of phages with carrier bacteria resulted in a significant reduction in disease incidence compared with the no phage control or the *P. agglomerans* carrier alone, and the protective effect was similar to that observed with streptomycin [17, 26]. 

Due to difficulties in isolating broad host range phages that infect suitable non-pathogenic strains, other strategies should be considered. For example, non-pathogenic mutant derivatives of the phytopathogen can be used as hosts for phage replication in the environment [40]. There are some risks if the pathogen is not fully attenuated or if reversion is possible. An alternative use for non-pathogenic or attenuated pathogenic hosts is for production of phages. Non-pathogenic hosts might enable safer and more economical phage amplification due to reduced purification requirements for removal of pathogenic bacteria. 

### 3.4. Different Plant Disease Systems

The disease symptoms and location of infection within or on the plant can pose challenges for phage biocontrol. For example, *Pseudomonas syringae *pv. *actinidiae* and *Erwinia amylovora* spend some of the disease cycle inside the host plant following infection through plant openings or wounds [4, 65]. High numbers of bacteria can accumulate within cankers in the plant and are protected from any control agent applied to the outside of the plant that cannot penetrate to deeper tissues. One study has shown a preventative and beneficial effect on disease progression with phage treatment of citrus canker [25]. However, the ability of phage to act as a curative agent of cankers was not directly assessed. High-pressure injection of phage is a possible strategy that is under consideration. In cases where treatment is problematic, phage prophylaxis would be the preferred application at a time when the chance of infection is greatest [17]. 

Phages survive in soil for at least one month [66] but the type of soil, pH, moisture content, and nutrients all influence persistence and can affect phage bioavailability [22, 67]. There is a desire to use phages in soil against bacterial pathogens that infect plant tubers, such as *Pectobacterium *spp. and *Dickeya* spp. In a recent study, treatment of potato tubers with phages prevented soft rot disease caused by *Dickeya solani* in a controlled environment [23]. Only a small protective effect was seen when the tubers were planted and only when the tubers were allowed to dry completely after treatment. A mixture of phages against *Dickeya solani* and *Pectobacterium atrosepticum* would be useful as Adriaenssens et al. observed soft rot caused by both species during their trials [23]. 

### 3.5. Use of Filamentous Phages

The filamentous phages *φ*RSS1 and *φ*RSM3 have almost opposite effects on the virulence of *Ralstonia solanacearum*. Infection of *Ralstonia solanacearum* with *φ*RSS1 increased twitching motility, EPS production, the expression of the virulence regulator *phcA*, and the rate of tomato plant wilting compared with a noninfected control [68]. In contrast, infection with *φ*RSM3 decreased twitching, EPS and *phcA* expression, growth, and movement in tomato plant stems and caused less wilting [69]. This difference was due to a repressor in the genome of *φ*RSM3 that is absent in *φ*RSS1 [69]. Tomato plants exposed to *φ*RSM3-infected bacterial cells increased the expression of genes to help the plant resist infection [28]. Indeed, pretreatment of tomato plants with *φ*RSM3-infected bacteria prevented infection by noninfected cells and prevented bacterial wilt. The authors suggest that a mixture of *φ*RSM3 and a lytic phage, such as *φ*RSL1 [41], would be a good BCA [28]. 

## 4. Application

Safety, efficacy, intellectual property, and a market are important factors for the commercial success of phage control of phytopathogens. As phages are ubiquitous and are consumed with food and water without any negative effects, their safety as BCAs is not an issue. Phages for control of *Listeria monocytogenes *have FDA approval for use on food [70] and have been granted GRAS (generally recognised as safe) status. Interestingly, in the EU [57] and the USA LISTEX (Micreos Food Safety) [71] is considered organic, suggesting that organic growers could be a valuable market for phage biocontrol products. Furthermore, phages are used to treat bacterial infections in humans in Russia and Georgia [72, 73]. Clinical trials have tested the effectiveness of phages to treat ear infections [74] and have assessed the safety of phages for treating burn victims [75]. These strictly controlled medical trials demonstrate that phage therapy is safe and effective, indicating that phage control of phytopathogens will not cause adverse health problems. Phages are being developed as BCAs for control of human pathogens in animals and on food products, which has been reviewed elsewhere [76, 77] and in other articles in this special issue. Patent protection and intellectual property are important factors in commercialisation of phage BCAs. Despite the concept of phage therapy having existed for over 90 years, multiple companies have acquired patents and established commercial platforms. This has been thoroughly reviewed recently by Gill et al. [78]. In the agricultural sector Omnilytics has developed AgriPhage, a range of phage products for the control of *Xanthomonas campestris *pv. *vesicatoria*, for the treatment of bacterial spot of tomatoes and peppers, and *Pseudomonas syringae *pv. *tomato*, which is the causative agent of bacterial speck on tomatoes. We expect further growth in the area of phage control of plant pathogens, which is the least examined application of phage control and has the advantage of less regulatory and safety hurdles.

## 5. Phage Resistance

**Figure 1:**
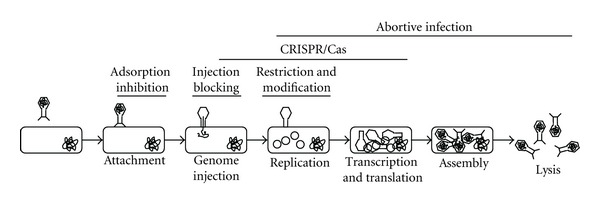
Bacteria can acquire phage resistance against most stages of the phage lifecycle. Fortunately, we can use our knowledge of these systems and the ability to evolve or isolate phage that infect resistant strains to minimise or avoid resistance (see text for details).

Bacterial mechanisms of phage resistance are well understood and should be considered when designing a BCA to reduce resistance and/or to help develop alternative BCAs. Most stages during phage infection can be affected by resistance development (Figure 1) [19, 79]. Briefly, these mechanisms include prevention of phage adsorption, blocking DNA entry, abortive infection, CRISPR/Cas systems, and restriction modification systems.

### 5.1. Receptors and Inhibition of Phage Adsorption

Cell surface receptors are essential for phage attachment. Receptor identification is important because receptor mutation is a common cause of phage resistance [80]. However, development of phage resistance can be beneficial. For example, mutants of *Pectobacterium atrosepticum *resistant to *φ*S32 had mutations in LPS, which reduced their virulence in a potato tuber rot assay [81]. Likewise, *Pectobacterium atrosepticum* mutants resistant to *φ*AT1 contained flagella mutations and were attenuated for motility and virulence [82]. The double-stranded RNA phages, *φ*6 and *φ*2954, that infect *Pseudomonas syringae* use Type IV pili for attachment [83–85]. Mutations in Type IV pili cause reduced survival in the phyllosphere and pathogenicity [86]. Phage *φ*6 also uses host cell phospholipids as secondary receptors during infection [87]. Because phages often recognize these important components (e.g., LPS and flagella), the resistant mutants are frequently less competitive or pathogenic. Therefore, careful choice of the phage cocktail can ensure that if resistant bacteria arise they will be attenuated. This approach has been utilised in phage therapy of *E. coli* infections in animal trials [88]. 

It is generally accepted that a phage mixture that uses different receptors is better for biocontrol because resistance to a carefully chosen range of phages cannot usually be acquired with a single point mutation [89, 90]. It is possible to use mutant bacterial hosts to enrich for the isolation of phages with alternative receptors. For example, a phage that targeted the flagellum was isolated using an LPS mutant host [82]. However, in most studies where multiple phages are used the exact receptors are unknown, limiting the potential benefit of such an approach. Cocktails are not always the most effective treatment. One study by Fujiwara et al. [41] characterised resistant strains generated by infection with three phages. Resistant strains were isolated for two phages, whereas no phage-resistant mutants were observed for a third phage. Use of this phage (*φ*RSL1) was more effective in tomato plant assays and greenhouse experiments compared with either of the other phages used singularly or as a mixture. Discovering the receptors used by these phages might shed some light on these results. To overcome resistance, Omnilytics has developed a management plan, which involves monitoring the pathogen and updating the phage mixture if, or when, bacterial resistance emerges [91]. This involves the selection of host range (h-) mutants, which can be evolved to avoid resistance [55]. 

### 5.2. Intracellular Factors and Abortive Infection/Toxin-Antitoxin Systems

Cytosolic factors are important for phage infection and their mutations can lead to resistance. *Pseudomonas syringae* phage *φ*2954 requires host glutaredoxin 3 (GrxC) for transcription of the third L segment of RNA and deletion of *grxC* led to resistance [92]. Phages with gene 1 mutations were easily selected that had overcome the loss of GrxC [92]. This indicates that phage can be isolated to overcome resistance caused by mutations of intracellular factors. Therefore, in theory, if *φ*2954 was to be used as a BCA, these escape mutants could be selected as part of the cocktail to avoid the impact of *grxC* mutants.

Other mechanisms interfere with phage reproduction such as the abortive infection (Abi) systems [19, 93]. Abi systems cause the “suicide” of infected cells and the inhibition of phage reproduction. Most Abi systems were identified in dairy bacteria but, recently, an Abi system, termed ToxIN, was isolated in *Pectobacterium atrosepticum *[94]. ToxIN inhibited infection by multiple phages and worked in different genera [94]. Theoretically, this broad efficacy and the presence of some of these systems on plasmids [94, 95] might pose a threat for phage as biocontrol agents. Fortunately, the only Abi system shown to function in a phytopathogen is ToxIN. ToxIN acts as a novel Type III protein-RNA toxin-antitoxin (TA) system [94, 96, 97]. TA systems consist of a toxic protein and an antitoxin and are found in most bacterial genomes [98]. Despite debated roles, TAs have been shown to provide resistance against phages [99]. Whether TA systems influence use of phage as biocontrol agents is at present unclear. Reassuringly, phage mutants can be isolated that avoid Abi/TA systems [97], demonstrating that resistance can be overcome if encountered. 

### 5.3. CRISPR/Cas Resistance

Recently, roughly 40% of sequenced bacteria have been shown to possess an adaptable phage resistance system [100]. These systems contain a clustered regularly interspaced short palindromic repeat (CRISPR) array and CRISPR associated (Cas) proteins. CRISPR arrays acquire short stretches of nucleic acids (termed spacers) from invading phages. The arrays are transcribed and processed into small RNAs, which, with the assistance of Cas proteins, target and degrade spacer-complementary viral nucleic acids. In short, CRISPR/Cas provides a heritable memory of past invaders and elicits a sequence-specific immunity. The experimental analysis of CRISPR/Cas systems in phytopathogens is limited to *Pectobacterium atrosepticum* [101], *Erwinia amylovora* [102], and *Xanthomonas oryzae* [103]. *Pectobacterium atrosepticum* has three CRISPR arrays and the *cas* operon is expressed *in planta* and *in vitro*, indicating that phage resistance could be active during plant infection [101]. In *Erwinia amylovora* the CRISPR/Cas was used to study the evolutionary history of these strains and some spacers matched viral sequences but not to any sequenced *Erwinia* phages [102]. In *Xanthomonas oryzae*, sequence analyses suggested that the system had previously provided resistance against phage Xop411 but the phage had acquired a mutation to avoid the resistance system [103]. The role of CRISPR/Cas systems in plant pathogens is not well characterised but phage can be selected to avoid CRISPR resistance if, and when, it arises [104]. In summary, despite the presence of multiple resistance mechanisms, our understanding of these systems and the ability to easily select phage escape mutants and create intelligent cocktails can minimise any possible impact of resistance development in an effective therapy. 

## 6. Conclusions and Future Directions

As plant diseases continue to have a serious impact on food production worldwide, new approaches for control are sought. This has seen a resurgence of studies into the use of phage for prophylaxis and treatment of phytopathogens. As highlighted in this paper, multiple phage-phytopathogen-plant systems have been studied and promising results are beginning to emerge. However, although available, commercial application of phages to treat plant disease is still uncommon. Alternative strategies for phage-based control of plant pathogens are being developed. For example, one idea is to insert phage genes into plant genomes, especially for the control of systemic pathogens. To avoid the requirement of producing large amounts of phages or purified enzymes, transgenic tomato plants that express the CMP1 and CN77 endolysins in the xylem are being developed to kill invading *C. michiganensis *[33, 34]. Despite the possible advantages of this approach, the regulatory approval needed because of the use of transgenic tomato plants may present a challenge in certain countries and to the consumers. In conclusion, studies into phage BCAs will not only aid in tackling the problems of plant diseases but will also continue to shed light on the basic biology of phages and their pathogenic bacterial hosts.

## Figures and Tables

**Table 1:** Phage therapy trials of plant pathogens.

Pathogen	Host	Disease		References
*Agrobacteriumtumefaciens*	Tomato	Crown gall	Bioassay with infected tissue—bacteriophage had no effect	[37]
*Dickeya solani*	Potato	Soft rot	Small effect seen when seed tubers were treated with phage prior to planting	[23]
*Erwinia amylovora*	Pome fruits	Fire blight	Phages isolated and characterised *in vitro*. Some testing *in planta*, promising results in combination with non-pathogenic carrier *Pantoea agglomerans *	[24, 38, 39]
*Pectobacterium carotovorum * subsp. *caratovorum *	Calla lily	Bacterial soft rot	Bacterial load reduction by phages but inhibition of killing by fertiliser solutions	[36]
*Ralstonia solanacearum*	Tobacco	Bacterial wilt	In a greenhouse trial pretreatment of plant roots with an avirulent strain and application of phage to the plants protected plants against bacterial wilt. No comparison was made to conventional chemical control methods	[40]
*Ralstonia solanacearum*	Tomato	Bacterial wilt	In a greenhouse trial pretreatment of tomato seedlings with *φ*RSL1 prevented bacterial wilt in all plants, untreated plants all wilted. *φ*RSL1 inhibited growth of bacteria but did not completely kill bacteria.	[41]
*Streptomyces scabies*	Potato	Potato scab	Phage treatment of seed tubers prior to planting reduced scab lesion coverage	[42]
*Xanthomonas arboricola* pv.* pruni *	Stonefruits	Bacterial spot	Application of phage to peach leaves prior to infection resulted in a 42% disease reduction compared to a nontreated control. Application of phage after infection had no effect	[43]
*Xanthomonas axonopodis* pv*. allii *	Onion	Xanthomonas leaf blight	Field and greenhouse trials of phage and plant activator provided equivalent protection to copper	[44]
*Xanthomonas axonopodis* pv.* vignaeradiatae *	Mungbean	Bacterial leaf spot	Synergistic effect of phage and streptomycin on mungbean seeds reduced seedling infection	[45]
*Xanthomonas campestris* pv.* juglandis *	Walnut	Walnut blight	Phage did not survive on walnut leaves in a greenhouse trial; pathogen was not included on leaves	[46]
*Xanthomonas campestris* pv.* pruni *	Peach	Leaf and fruit spot	A significant reduction in disease was seen in one out of three orchards with weekly application of a single phage	[47]
*Xanthomonas campestris* pv.* vesicatoria *	Tomato and Pepper	Bacterial spot	These studies have led to the successful development of a phage BCA (http://www.omnilytics.com/).	[48–51]
*Xanthomonas citri *subsp*. citri *	Citrus	Citrus canker	Mixed results in greenhouse and nursery trials when compared to copper bacteriocides	[25]
*Xanthomonas fuscans *subsp.* citrumelonis *	Citrus	Citrus bacterial spot	Mixed results in greenhouse and nursery trials when compared to copper bacteriocides	[25]
